# Testicular Torsion in a Rare Case of Prostatic Spindle Cell Rhabdomyosarcoma

**DOI:** 10.7759/cureus.12238

**Published:** 2020-12-23

**Authors:** Subramaniyan Ashuvanth, Ramanan Sinduja, Chellappa Vijayakumar, Uday Kumbhar, Pampa C Toi

**Affiliations:** 1 Surgery, Jawaharlal Institute of Postgraduate Medical Education and Research (JIPMER), Puducherry, IND; 2 Patholoy, Jawaharlal Institute of Postgraduate Medical Education and Research (JIPMER), Puducherry, IND

**Keywords:** testicular torsion, epidiymo-orchitis, prostatomegaly, spindle cell, rhabdomyosarcoma

## Abstract

Spindle cell sarcoma of the prostate is a rare variant of rhabdomyosarcoma (RMS), with the clinical experience limited to a handful of cases in the literature. We report a rare case of spindle cell rhabdomyosarcoma (SC-RMS) who presented to us with testicular torsion features. A 20-year-old male presented initially with lower urinary tract symptoms and features of epidiymo-orchitis, which was managed conservatively. He later presented with features suggestive of testicular torsion and underwent orchidopexy. The patient developed postoperative urinary retention. On per-rectal examination, there was gross prostatomegaly. He was evaluated with a trans-rectal biopsy and contrast-enhanced computed tomography (CECT). Subsequently, he was found to have metastatic prostatic SC-RMS, which is a rare disease. He was started on chemotherapy and radiotherapy. SC-RMS is a variant that has a comparatively favorable prognosis in children and an aggressive course in adults, as reported in the few cases so far. The treatment regimen is still evolving, and there is no consensus on the therapeutic approach. All these factors together result in the dismal outlook of patients diagnosed with this condition.

## Introduction

Skeletal muscle soft tissue tumor includes rhabdomyoma and rhabdomyosarcoma (RMS). These are common in infants and children and reported very rarely in adults. The spindle cell variety is a rare variant of the embryonal RMS. Any part of the body can be affected by RMS. However, the most common primary sites are the head and neck region (35-40%), the genitourinary tract (25%), and the extremities (20%) [1]. The disease presents with a pressure effect in the affected regions. A surgical emergency in case of testicular torsion occurs more often in neonates and young adolescent populations. The incidence of testicular torsion peaks at neonatal and puberty period. The estimated incidence of testicular torsion in Asian men is 3.5 cases per 100,000 under 25 years [2]. It can result in the loss of the testicle if not intervened promptly. The prognosis in this condition greatly depends upon the time of intervention from the insult. Best results are reported when the exploration and restoration of flow are achieved within six hours of ischemia initiation. We present such a rare case of spindle cell rhabdomyosarcoma (SC-RMS) of the prostate in a 20-year-old male who presented to us with symptoms of testicular torsion.

## Case presentation

A 20-year-old male presented to surgical emergency with complaints of acute onset throbbing pain of the left hemiscrotum for one day. He had no associated fever. He also had difficulty defecating and a sense of rectal fullness that had gradually increased over one month. The patient had previously presented with acute urinary retention features three weeks before this episode, for which he was catheterized. He was then evaluated by the urology team, who found an enlarged prostate. The patient’s urinalysis showed plenty of red blood cells (RBCs), traces of protein with no pus cells on their further evaluation. An ultrasonogram (USG) done at that time showed an enlarged prostate with thickened left side cord structures and increased vascularity, which was suggestive of prostatitis and left epididymo-orchitis with funiculitis. He was then managed conservatively with oral antibiotics.

**Figure 1:**
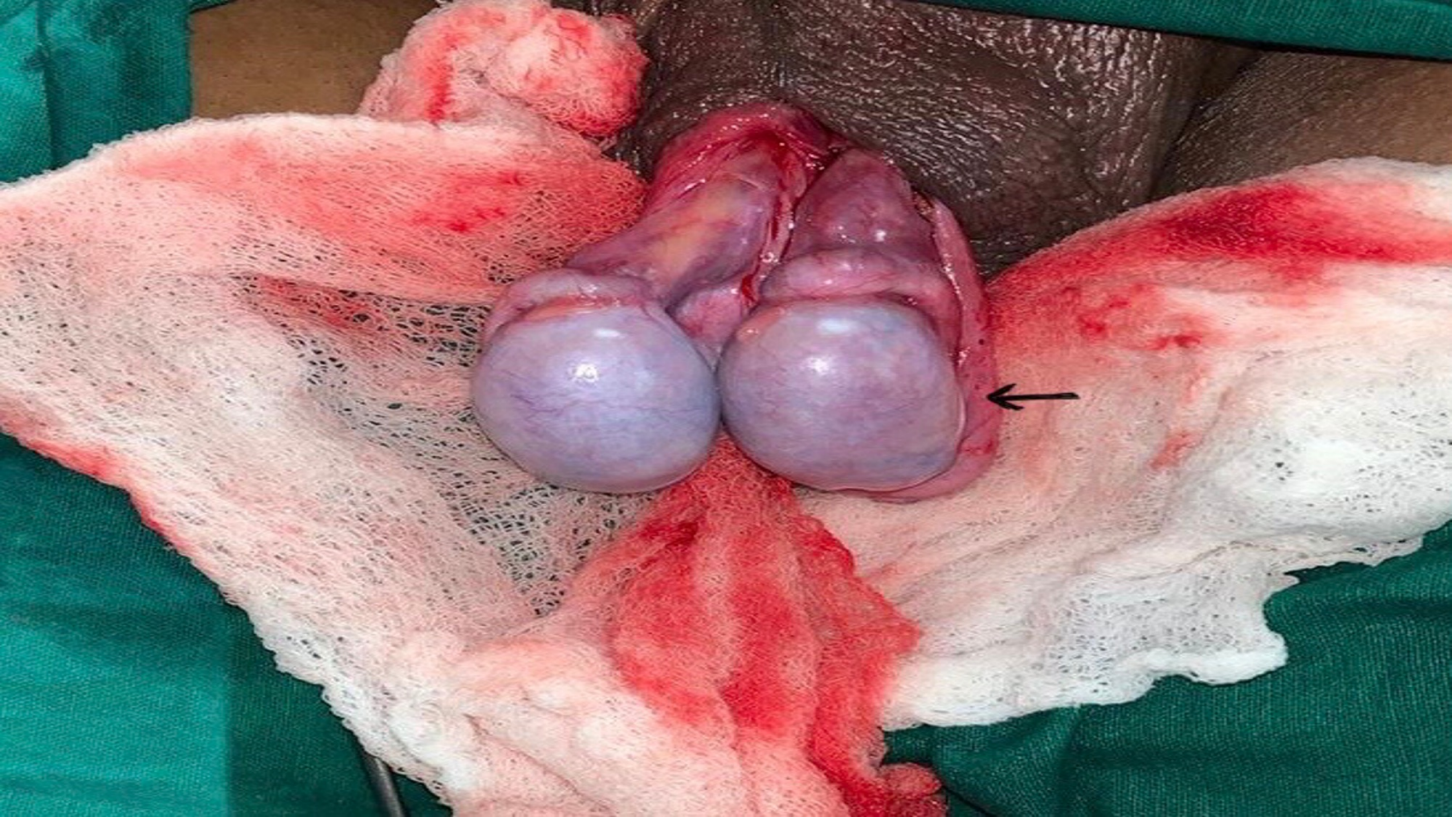
Intraoperative picture of torsion testis with mildly dusky left testis (arrow)

The patient had mild lower abdominal pain, and on examination, there was tenderness over the left hemi-scrotum and lower abdomen. There was no local warmth or redness. The cord structures on the left side were tender and bulky. Right side testis and cord structures were normal on examination. On elevating the left testis, the pain was aggravated, suggesting a clinical diagnosis of testicular torsion. On per rectal examination, a grossly enlarged prostate, almost occluding the lumen, was noted. An USG examination of the scrotum was strongly suggestive of compromised vascularity to the left testis, and the patient was taken up for emergency scrotal exploration. It revealed a dusky left testis and twisted left cord. The cord was retorted, and orchidopexy was done bilaterally. Postoperatively there was an improvement in the testicular pain, and the patient was symptomatically better (Figure 1).

**Figure 2:**
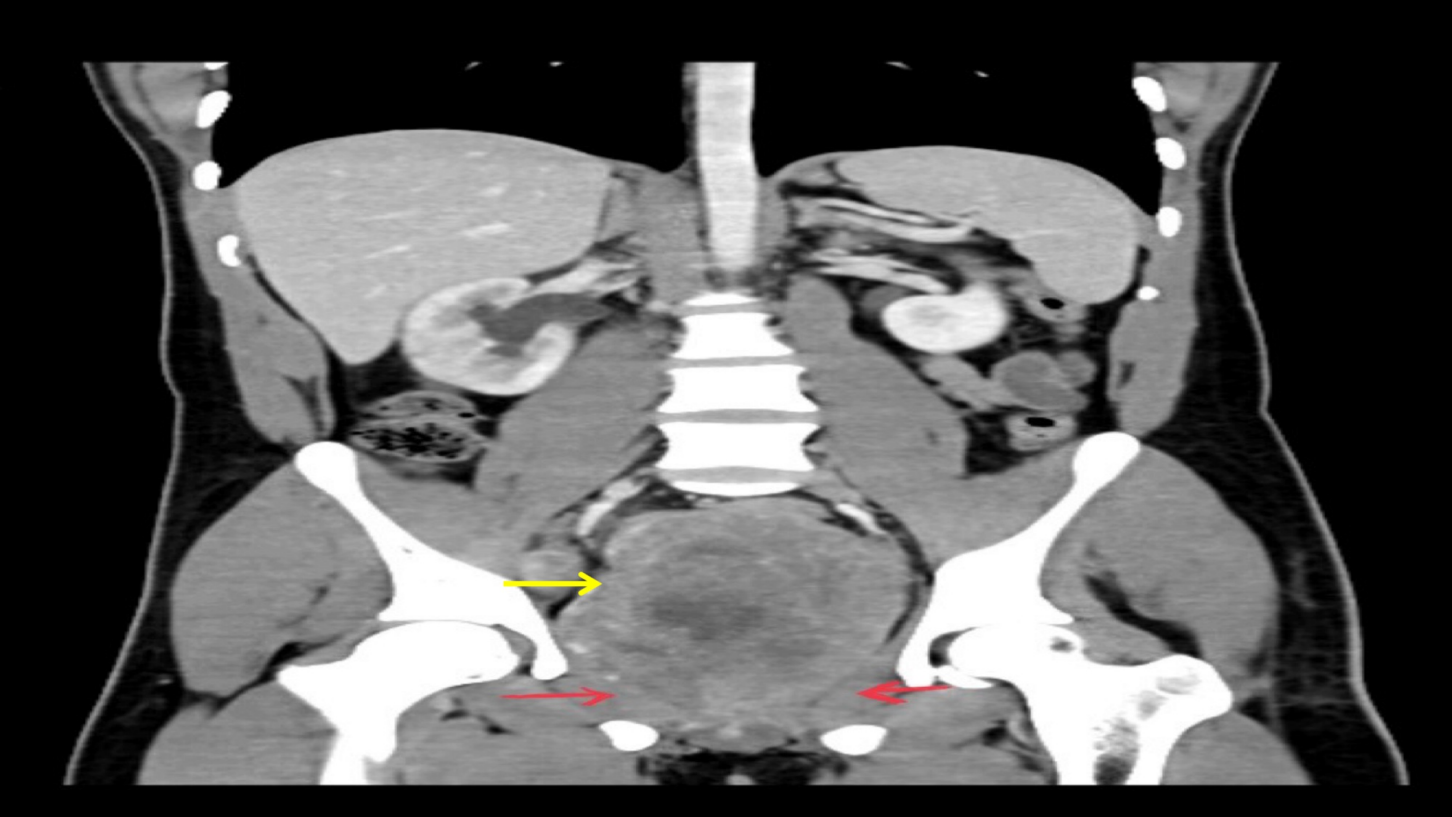
CECT showing the prostate gland replaced by a mass (yellow arrow) that seems to infiltrate the lateral pelvic wall (red arrows) CECT - contrast-enhanced computerized tomography

A contrast-enhanced computerized tomography (CECT) with an excretory phase (CT urography) was done to evaluate the grossly enlarged prostate gland. It showed that the prostate was replaced by a heterogeneous enhancing large irregular lesion, measuring approximately 8.5 x 9.9 x 10.1 cm (Figure 2).

**Figure 3:**
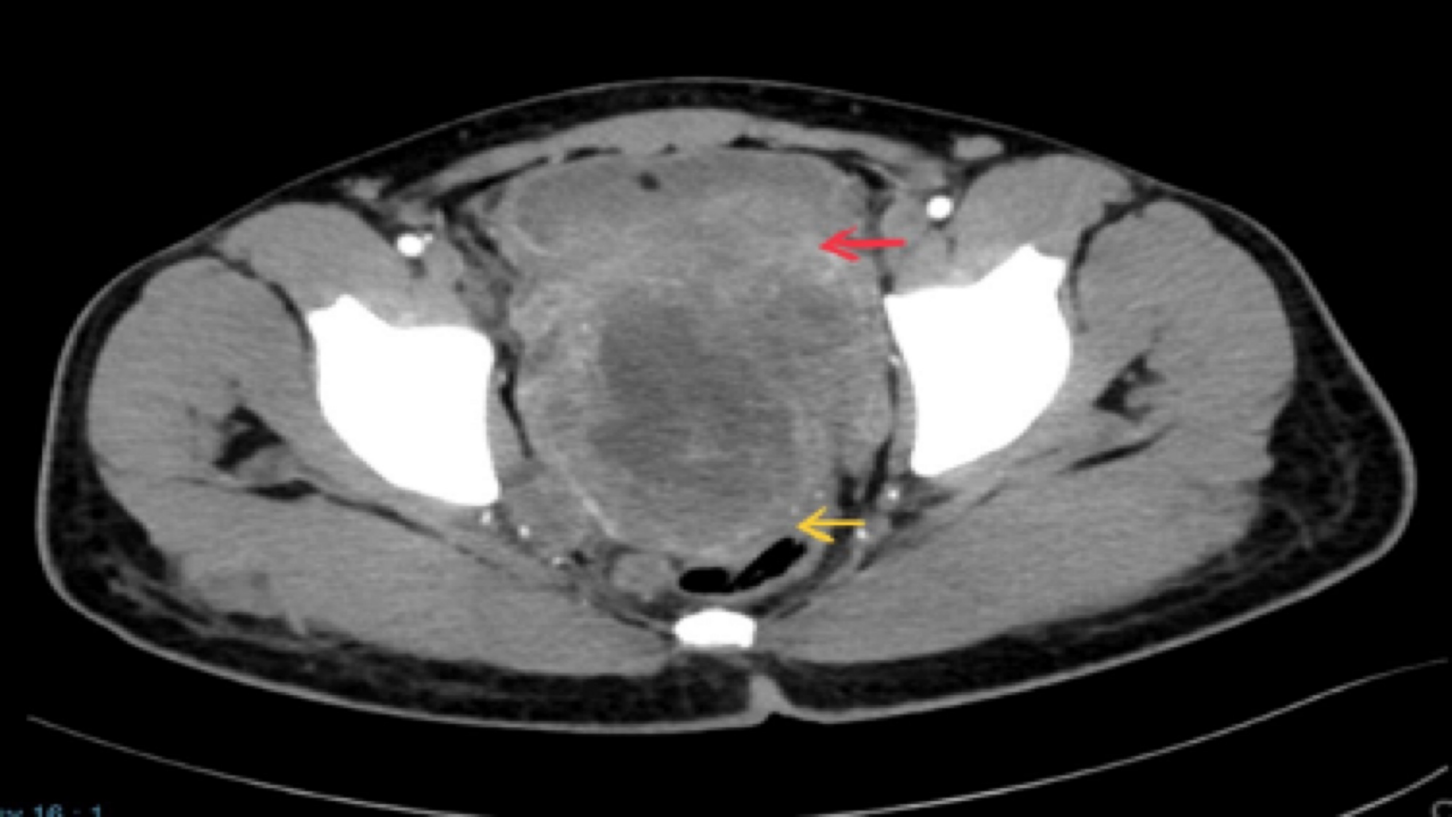
CECT showing the tumor infiltrating the rectum (yellow arrow) posteriorly and bladder (red arrow) anteriorly CECT - contrast-enhanced computed tomography

Anteriorly, it infiltrated the posterior wall of the urinary bladder and projected into the lumen. It was encasing the prostatic urethra with the loss of fat planes. Laterally, it reached up to the lateral pelvic wall with loss of fat planes with bilateral obturator internus muscles. It showed loss of fat planes with the rectum, displacing it posteriorly (Figure 3).

Inferiorly it had lost fat planes with the levator ani. Heterogeneously enhancing bilateral internal iliac, right external iliac, and perirectal lymph nodes were noted. The features were suggestive of locally advanced prostatic malignancy. Since the patient’s renal parameters were within normal limits and the cause for bilateral hydroureteronephrosis (HUN) was downstream, he was managed with per urethral catheterization to relieve the obstruction at the level of the prostate. 

**Figure 4:**
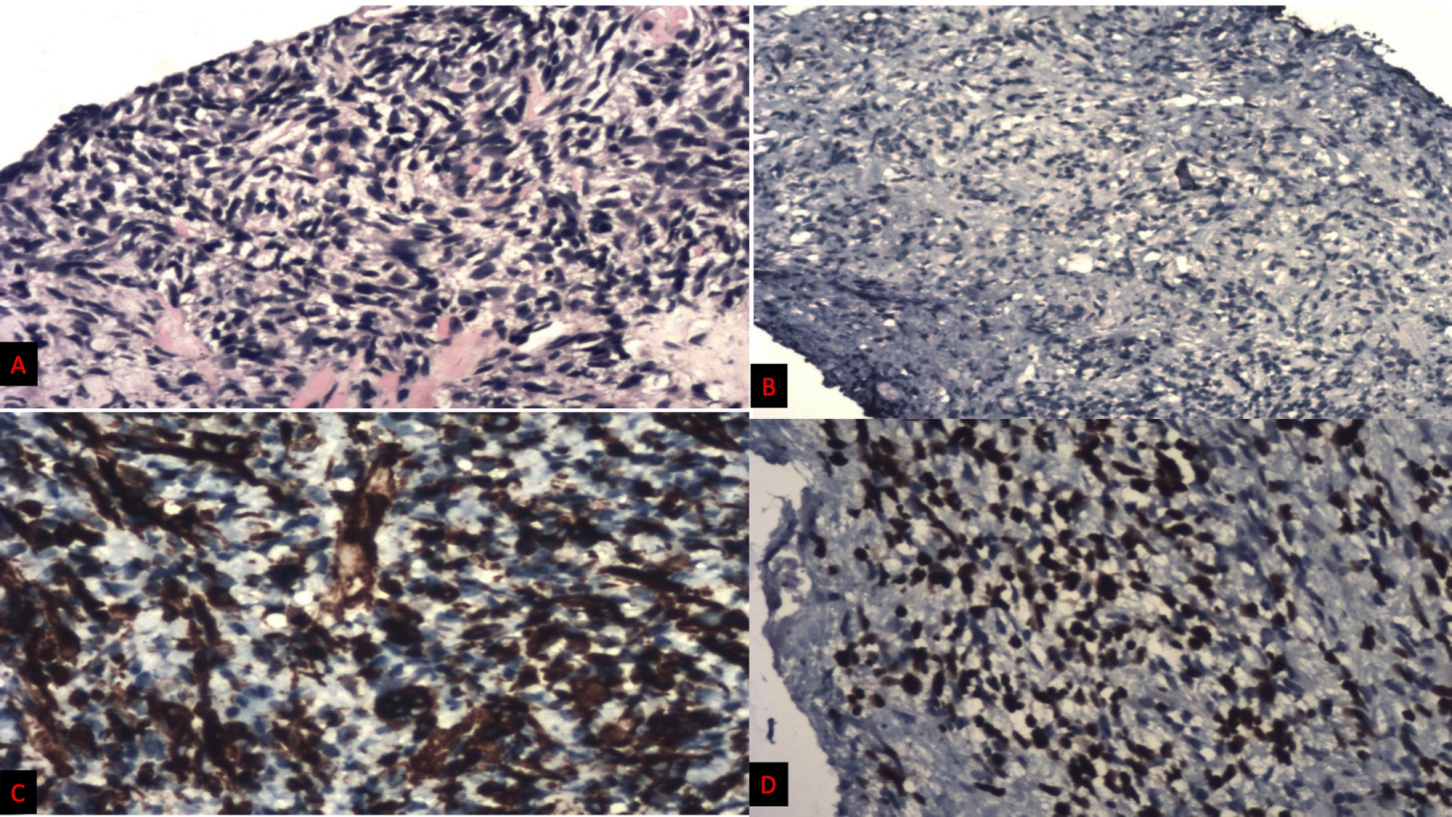
Microscopic examination of trans-rectal biopsy from the prostate A) Histology showing sheets of spindle cells. B) IHC staining showing PSA negativity. C) IHC showing desmin strongly positive. D) IHC showing Myo-D1 strongly nuclear positive. IHC - immunohistochemistry; PSA - prostate-specific antigen

The patient then underwent a trans-rectal biopsy from the prostate. Histopathological examination (HPE) of the biopsy showed scores of fibromuscular tissue infiltrated by a tumor composed of sheets and fascicles of spindle cells displaying mild to moderately pleomorphic, ovoid to elongated hyperchromatic nuclei, inconspicuous nucleoli, and scant eosinophilic cytoplasm. On immunohistochemistry (IHC), the tumor cells were diffusely and strongly positive for desmin and Myo-D1 and were negative for cytokeratin (CK), smooth muscle actin​​​​​​​ (SMA), Schwannian markers (S 100 protein), synaptophysin, chromogranin, CD34, and prostate-specific antigen (PSA). The features were suggesting a diagnosis of RMS of the spindle cell type (Figure 4).

Further, a whole-body positron emission tomography CT (PET-CT) scan was done. Increased metabolic activity was noted in the right external iliac, bilateral internal iliac, a few presacral, and left inguinal lymph nodes. The scan also revealed increased fluorodeoxyglucose (FDG) uptake in segment VI of the liver, T10, L1-L2 vertebrae, the sacrum, and the neck of the right femur. Hence a diagnosis of metastatic SC-RMS was made. During his hospital stay, he developed frank hematuria through the per-urethral catheter. USG of the abdomen revealed hematoma in the urinary bladder. He was managed conservatively with bladder irrigation, which settled the hematuria. Bladder tumors may be associated with hematuria and urinary obstruction, while prostatic primaries typically present as large pelvic masses causing urinary frequency or constipation from extrinsic compression of the bladder or intestinal tract. However, the hematuria, in this case, was due to the invasion of the bladder by the tumor. His performance status rapidly deteriorated, and he was confined to bed most of his waking hours. A multidisciplinary team (MDT) discussion was done, and he was started on chemotherapy with vincristine, actinomycin-D, and cyclophosphamide regimen (VAC regimen). He was given the first cycle of chemotherapy, which he tolerated well. He was discharged and received chemotherapy on a daycare basis. Following that, he received radiotherapy - 60Gy in 30 fractions. The patient subjectively noticed some improvement in pain and constipation; however, he continued to have rectal fullness. He also developed symptoms of peripheral neuropathy that was suspected to be due to vincristine. His performance status continued to be poor, and he is bedridden for most of the day.

## Discussion

SC-RMS is a rather rare disease affecting infants and children. Among the various soft tissue sarcoma of childhood and adolescence, RMS is the commonest type with skeletal muscle differentiation [3]. There are three types of RMS based on histology: embryonal, alveolar, and pleomorphic RMS. The spindle cell variety is a rare variant of the embryonal RMS. This variant contributes to only 3% of all RMS [4]. The incidence in adults is even rarer, and clinical experience with the disease is limited. A literature search revealed a total of 30 cases of prostatic RMS in adults reported until now. This spindle cell variant has a predilection for younger males, and they are commonly seen affecting the paratesticular regions and the head and neck region. This particular subtype has a better prognosis in children compared to other variants of RMS [5]. In contrast, this variant is very aggressive in the adult population, associated with poor prognosis [6]. Also, Myo-D1 mutation-positive tumors were associated with an even worse prognosis [7]. A high degree of suspicion is essential to diagnose this condition, especially in adults, in whom the prognosis is bad, as the tumor is aggressive. So early diagnosis and instituting of multimodality therapy may aim at increasing the survival of these patients. 

The diagnosis of SC-RMS of the prostate can be challenging, as in our case, where it was initially diagnosed as a case of prostatitis, especially considering the younger age at which this tumor had presented. The patient was neither too young to consider RMS nor too old to consider prostatic carcinoma as a diagnosis. Also, he initially presented with a picture of epididymo-orchitis with prostatitis. This could have been, in fact, due to the infection resulting from the urinary stasis that had occurred due to the prostatic tumor. Also, that might have been overlooked, considering the age of the patient. However, on further evaluation, he was found to have a prostatic tumor, which was SC-RMS. There can also be a dilemma in diagnosing this condition in histology, as the histological picture can closely resemble other conditions. HPE of the SC-RMS consists of interspersed fascicles of elongated spindle cells. These cells may occasionally be arranged in whorls or in a herringbone pattern that is seen with leiomyosarcoma. And so it has been previously described as "leiomyomatous" embryonal RMS. Significant confusion occurs between the smooth muscle neoplasms and the SC-RMS due to their resemblance to histology. This is where the IHC can be of help in diagnosing the condition. The tumor's primary site, the nodes involved, and the metastatic sites are all the target sites for irradiation. The outlook with these therapeutic agents is encouraging in children with limited disease. However, in adults, the experience is limited, and outcomes are poor. 

As our patient was evaluated for the prostatic mass with CECT and biopsy, SC-RMS diagnosis came to light. We suspected that the symptom of testicular pain could be due to compression of the left renal vessels or testicular vessels by the retroperitoneal nodes.** **Because the clinical symptoms were very similar to testicular torsion, and the USG showed a definitive reduction of flow to the testis, the patient was explored. However, intra-operatively, there was duskiness of the left testis. SC-RMS is one of the types of sarcoma that can have nodal metastasis. On CECT, there were a few retroperitoneal nodes; however, there were no significant nodes that had any pressure effect or invasion of the testicular or renal vessels. The fact that the patient had relief from the acute testicular pain postoperatively also questions our theory. The renal vessel doppler was also done as a part of the hypertension workup and did not reveal any flow abnormality. So the above theory was discarded, and we finally concluded that it was, in fact, coincidental that our patient had testicular torsion with SC-RMS. The literature search did not reveal any similar case reports that showed an association between testicular torsion and SC-RMS. So we present this case report to document this occurrence of SC-RMS with testicular torsion so that a similar presentation in the future can help reveal the relation between the two entities, if any.

## Conclusions

SC-RMS is a rare neoplasm that affects children and neonates. Few case reports have been reported in adults, in whom the disease is quite aggressive, with a poor prognosis. As described in this report, there can be a delay in diagnosing this malignancy in a young adult. We hereby report such a case of SC-RMS in a young adult who also had symptoms suggestive of testicular torsion, which could not be explained by the primary disease itself. We concluded it to be a coincidental disease process. Any more similar presentations in the future may throw light on the association between the two conditions, which we could not confirm at this stage.
